# Langer mesomelic dysplasia as a rare manifestation of *SHOX* deficiency: a narrative review

**DOI:** 10.3389/fgene.2026.1876732

**Published:** 2026-07-22

**Authors:** Hubert Piwar, Jan Pawlasek, Patryk Sielaff, Maria Sztachelska, Michal Ordak

**Affiliations:** 1 Department of Pharmacotherapy and Pharmaceutical Care, Faculty of Pharmacy, Medical University of Warsaw, Warsaw, Poland; 2 Faculty of Medical Sciences, Warsaw Medical Academy of Applied Sciences, Warsaw, Poland; 3 Centre of Regenerative Medicine, Medical University of Bialystok, Bialystok, Poland

**Keywords:** Langer mesomelic dysplasia, mesomelic skeletal dysplasia, rare disease, short stature, SHOX deficiency

## Abstract

Langer mesomelic dysplasia is an exceptionally rare skeletal dysplasia caused by complete or functionally complete deficiency of the *SHOX* (short stature homeobox) gene located within the pseudoautosomal region 1 (PAR1) of the sex chromosomes. Clinically, the disorder is characterized by severe disproportionate short stature and marked mesomelic shortening of the limbs, particularly involving hypoplasia or aplasia of the ulna and fibula, while cognitive development and life expectancy are generally preserved. This narrative review summarizes current knowledge regarding the molecular genetics, developmental biology, clinical manifestations, radiographic findings, prenatal diagnosis, and differential diagnosis of Langer mesomelic dysplasia. The SHOX protein functions as a homeodomain-containing transcription factor essential for chondrocyte proliferation, differentiation, and growth plate organization. Pathogenic mechanisms include biallelic *SHOX* deletions, enhancer-region defects, missense variants affecting the homeodomain and nuclear localization signal, as well as splice-site variants leading to severe reduction of functional protein dosage. The article also discusses the broad phenotypic spectrum of *SHOX* deficiency, genotype-phenotype variability, and the relationship between Langer mesomelic dysplasia and related disorders such as Léri-Weill dyschondrosteosis and Turner syndrome. Understanding the molecular basis of this condition is essential for accurate diagnosis, genetic counseling, and prenatal assessment in affected families.

## 1 Introduction

Langer mesomelic dysplasia (LMD; OMIM #249700) was first characterized as a distinct clinical entity in 1967 (Langer, 1967). This rare skeletal dysplasia is defined by severe disproportionate short stature and pronounced mesomelic shortening of the limbs, typically involving hypoplasia or aplasia of the ulna and fibula (Langer, 1967; Kunze and Klemm, 1980). While the phenotype involves significant skeletal malformations, cognitive development and life expectancy are typically unaffected (Langer, 1967; Aggarwal et al., 2014). LMD belongs to the mesomelic dysplasias, a group of genetic disorders primarily affecting the middle (mesomelic) segments of the extremities (Langer, 1967). To date, LMD remains an exceedingly rare condition, with fewer than one hundred cases documented in the medical literature (Aggarwal et al., 2014; Fukami et al., 2016).

LMD results from biallelic inactivation of the *SHOX* gene (Short Stature Homeobox-containing gene) (Blaschke and Rappold, 2000; Zinn et al., 2002; Fukami et al., 2016). The clinical manifestations of *SHOX* deficiency encompass a broad phenotypic spectrum, ranging from mild idiopathic short stature (ISS) to severe skeletal malformations, including Léri-Weill dyschondrosteosis (LWD; OMIM #127300) (Blaschke and Rappold, 2000; Fukami et al., 2016). As a genetically related biallelic *SHOX* disorder, LMD represents the severe homozygous or compound-heterozygous counterpart of LWD (Kunze and Klemm, 1980; Zinn et al., 2002).

Pathogenic variants of the *SHOX* gene include whole or partial deletions or duplications, sequence variants within the coding region, and deletions involving upstream or downstream regulatory elements (Marchini et al., 2007b; Fukami et al., 2016). Deletions of the partial or entire *SHOX* locus or its downstream enhancers account for the majority of identified pathogenic alterations. The remaining fraction comprises intragenic coding-sequence and splice-site variants, including missense, truncating, and splice-disrupting variants, many of which affect the homeodomain or normal *SHOX* transcript processing (Zinn et al., 2002; Sabherwal et al., 2004a; Marchini et al., 2007b; Fukami et al., 2016; Gürsoy et al., 2020; Kohkalani et al., 2025). The homeodomain is critical for the protein’s DNA-binding and nuclear localization functions (Sabherwal et al., 2004b; Marchini et al., 2007b).

An extensive review of the available literature was conducted using major biomedical databases, including PubMed, Scopus, ScienceDirect, and Google Scholar, to identify publications related to Langer mesomelic dysplasia and SHOX deficiency. The evaluated studies included clinical reports, genetic analyses, radiological descriptions, and molecular investigations concerning the biological role of SHOX in skeletal development. Particular attention was directed toward the phenotypic spectrum of SHOX-related disorders, mechanisms of pathogenic variants, genotype–phenotype variability, and prenatal diagnostic findings. Information obtained from published case reports, family studies, and cohort analyses was critically assessed and incorporated into a comprehensive overview of the current knowledge regarding the molecular and clinical aspects of Langer mesomelic dysplasia.

## 2 SHOX gene structure and function

### 2.1 Genomic organization

The *SHOX* gene is located in the pseudoautosomal region 1 (PAR1) of both the X and Y chromosomes (Rao et al., 1997). It characteristically escapes X-chromosome inactivation during embryogenesis; consequently, two functional copies are essential for normal skeletal development (Blaschke and Rappold, 2000; Durand et al., 2011). The genomic structure of *SHOX* comprises seven exons and multiple evolutionarily conserved non-coding regulatory elements distributed upstream and downstream of the gene, which collectively coordinate its complex transcriptional regulation (Fukami et al., 2016; Spurna et al., 2022). Several *SHOX* transcripts exist due to alternative splicing, with two primary isoforms predominating: the longer *SHOXa* transcript and the shorter *SHOXb* variant (Marchini et al., 2007b; Durand et al., 2011).

**FIGURE 1:**
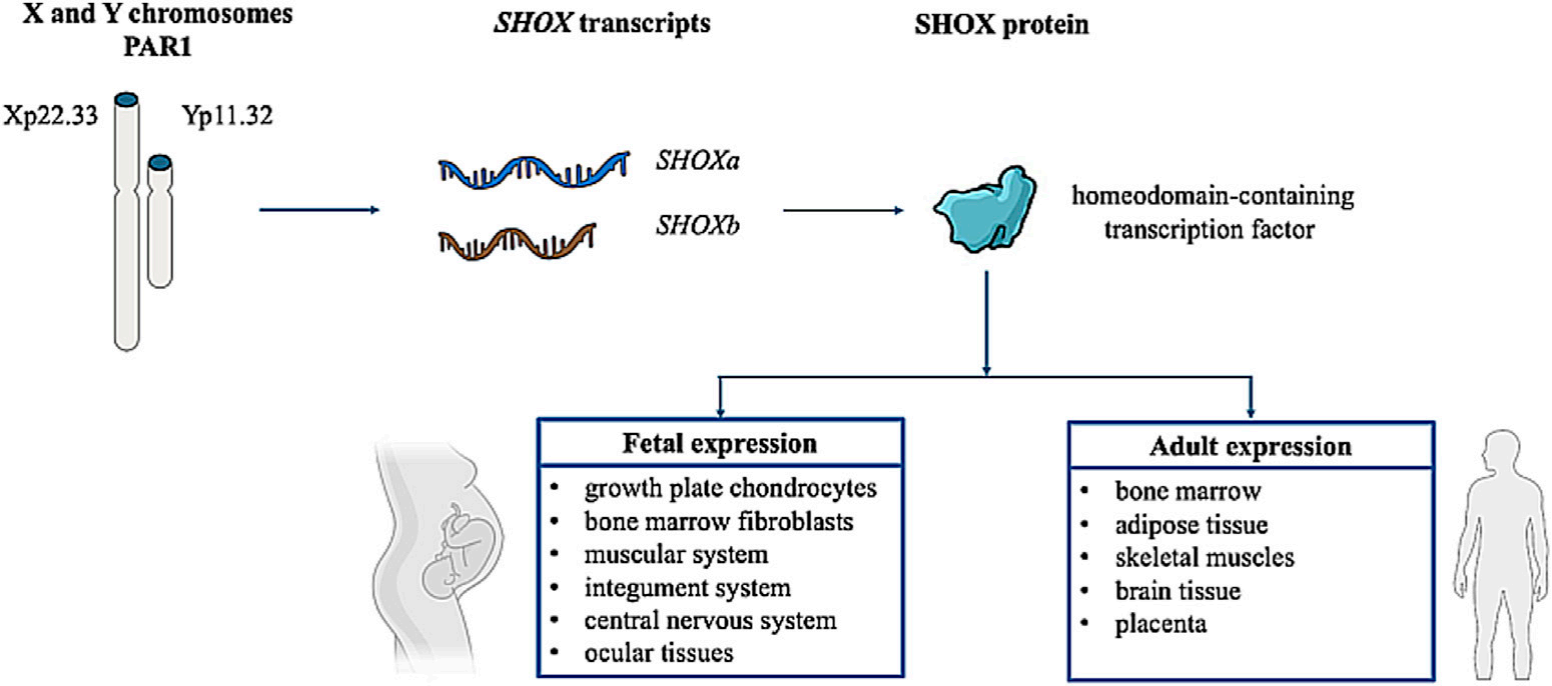
*SHOX* gene structure, transcripts, and tissue expression patterns. The *SHOX* gene resides in the pseudoautosomal region 1 (PAR1) at the tip of both sex chromosomes (Xp22.33 and Yp11.32). Two main alternative splice isoforms (*SHOXa* and *SHOXb*) encode a homeodomain-containing transcription factor. *SHOX* shows broad fetal expression, including growth plate chondrocytes, bone marrow fibroblasts, muscular system, integument, central nervous system, and ocular tissues, and more restricted adult expression in bone marrow, adipose tissue, placenta, skeletal muscle, and brain (based on Marchini et al., 2007b; Gahunia et al., 2009; Durand et al., 2011; author-prepared schematic using freely reusable illustrations from NIAID NIH BioArt Source, bioart.niaid.nih.gov/bioart).

Figure 1 summarises the genomic location of *SHOX* in PAR1, its two main alternative isoforms (*SHOXa* and *SHOXb*), and the contrast between broad fetal and more restricted adult expression patterns.

### 2.2 Protein function in skeletal development

The *SHOX* gene encodes a homeodomain-containing transcription factor that functions as a key regulator of longitudinal skeletal growth (Marchini et al., 2007b). Its expression is initiated during early embryonic development, primarily within growth plate chondrocytes and bone marrow fibroblasts (Marchini et al., 2007b; Gahunia et al., 2009; Seki et al., 2014). Although some reports have also described *SHOX* expression in trabecular osteoblasts (Gahunia et al., 2009), immunohistochemical studies of the growth plate did not confirm expression in osteoblasts or osteoclasts (Fukami et al., 2016). In the postnatal period, *SHOX* expression remains localized to the epiphyseal growth plates (Gahunia et al., 2009). Fetal *SHOX* expression is broad and includes the musculature, integument, central nervous system, and ocular tissues, whereas adult expression is more restricted, persisting in bone marrow, adipose tissue, placenta, skeletal muscle, and brain (Durand et al., 2011).

The SHOX protein participates in the regulation of aggrecan synthesis through direct interactions with SOX5 protein and SOX6 protein, members of the SOX trio that, together with SOX9 protein, coordinate chondrogenesis and the assembly of the cartilage matrix (Aza-Carmona et al., 2011). The SHOX protein also directly induces transcription of the *NPPB* gene, encoding the natriuretic peptide BNP, a direct *SHOX* target whose co-expression with *SHOX* in prehypertrophic and hypertrophic chondrocytes implicates this axis in growth plate regulation (Marchini et al., 2007a). These interactions are critical for the precise control of chondrocyte proliferation and differentiation within the epiphyseal growth plate.

SHOX protein-mediated apoptosis has been implicated in the modulation of chondrocyte hypertrophy. In osteosarcoma cell models, the overexpression of SHOX protein has been shown to elicit oxidative stress, leading to lysosomal membrane rupture and the initiation of cathepsin B-dependent apoptotic pathways. These findings support the hypothesis that SHOX protein regulates cell death processes during endochondral ossification (Hristov et al., 2014).

**FIGURE 2:**
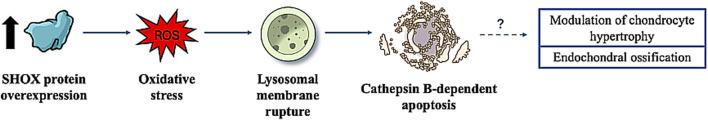
Proposed model of SHOX-mediated apoptosis pathway in chondrogenesis. In experimental models using osteosarcoma cell lines (Hristov et al., 2014), overexpression of SHOX protein induces oxidative stress through accumulation of reactive oxygen species (ROS), leading to lysosomal membrane rupture and release of cathepsin B into the cytoplasm, which initiates apoptosis. The dashed arrow indicates the hypothetical translation of these *in vitro* findings to the *in vivo* regulation of chondrocyte hypertrophy and endochondral ossification. Author-prepared schematic using freely reusable illustrations from NIAID NIH BioArt Source (bioart.niaid.nih.gov/bioart).

Figure 2 depicts the proposed cascade of events linking SHOX protein overexpression, oxidative stress and lysosomal cathepsin B-dependent apoptosis as observed in osteosarcoma cell models.

The SHOX protein has been identified as a complex transcriptional regulator of *FGFR3* gene expression, exhibiting cell-type-specific effects. While the SHOX protein positively regulates *FGFR3* expression in normal human dermal fibroblasts, it exerts a repressive effect in chicken micromass cultures, a model that mimics endochondral ossification and embryonic limb development. Given that the FGFR3 protein is a known negative regulator of endochondral bone growth, this SHOX protein-mediated repression is likely critical for growth homeostasis in mesomelic segments. Consequently, *SHOX* deficiency may lead to a relative increase in *FGFR3* expression, which could contribute to the skeletal phenotype observed in affected individuals (Decker et al., 2011).

**FIGURE 3:**
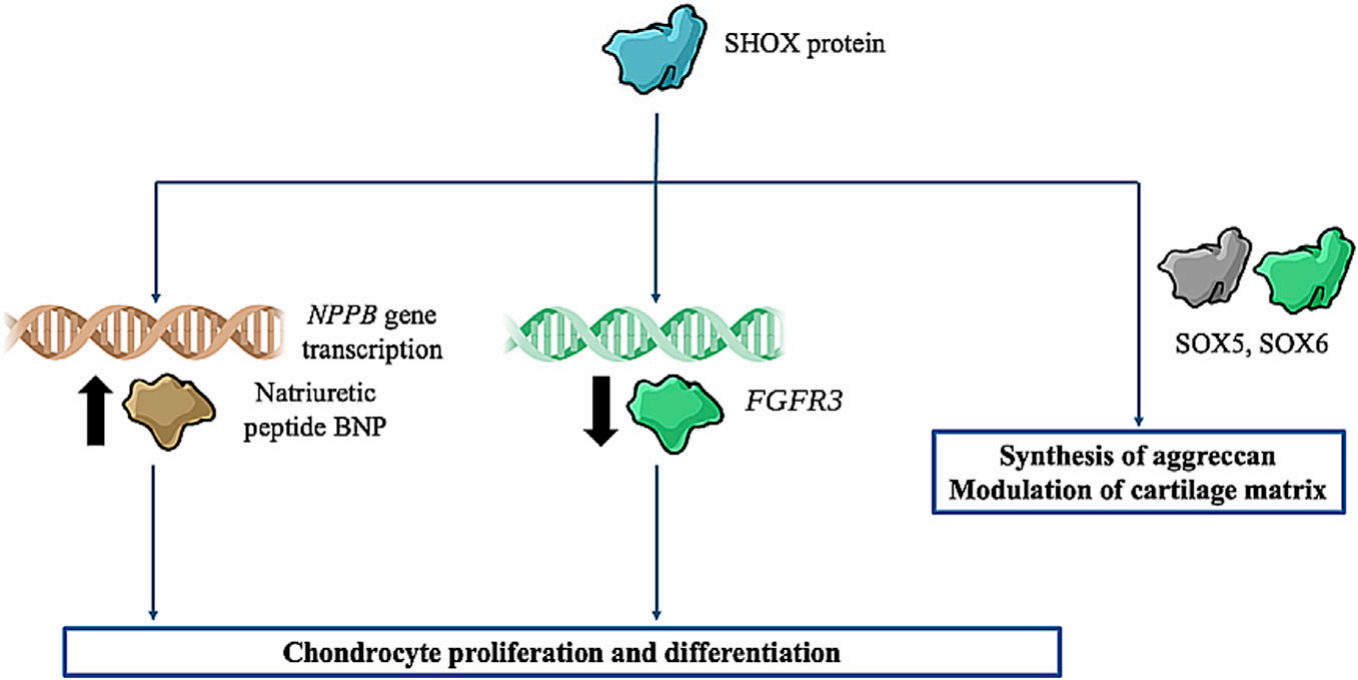
SHOX-mediated regulation of chondrogenesis. Schematic representation of major downstream targets of SHOX protein in the chondrogenic environment. SHOX protein upregulates *NPPB* gene transcription, leading to BNP production; represses *FGFR3* expression in the chondrogenic context; and interacts directly with SOX5 protein and SOX6 protein to regulate aggrecan synthesis and cartilage matrix assembly. Together, these pathways regulate chondrocyte proliferation, differentiation, and cartilage matrix organization. SHOX protein exerts cell-type-specific regulation of *FGFR3*; the depicted repression reflects the chondrogenic context. Author-prepared schematic using freely reusable graphical elements from NIH/NIAID BioArt Source (bioart.niaid.nih.gov/bioart).

Figure 3 schematically integrates these chondrogenic regulatory axes, illustrating SHOX protein-mediated upregulation of the *NPPB* gene (encoding BNP), repression of *FGFR3* in the chondrogenic context, and direct interactions with SOX5 protein and SOX6 protein to modulate aggrecan synthesis and cartilage matrix assembly.

In human embryos, *SHOX* and *SHOX2* display complementary expression profiles during limb development: *SHOX* is detected in the middle portion of the limb corresponding to the mesomelic segments, whereas *SHOX2* is expressed more proximally (Cobb et al., 2006; Marchini et al., 2007b). This spatial partitioning likely explains the selective vulnerability of the zeugopod (forearm and lower leg) in *SHOX*-deficient conditions such as LWD and LMD, as the proximally expressed *SHOX2* cannot compensate for *SHOX* loss in distal segments (Cobb et al., 2006). Consistently, conditional *Shox2* inactivation in murine limbs, a species that has lost the *SHOX* gene during evolution, leads to severe stylopodial (humerus, femur) shortening, with relative sparing of the zeugopod, confirming the complementary segmental roles of these paralogs (Cobb et al., 2006; Yu et al., 2007). The murine Shox2 protein shares 79% amino acid identity with human SHOX protein, including an identical DNA-binding homeodomain (Cobb et al., 2006). In the murine model, Shox2 has been shown to regulate chondrogenesis during the early stages of differentiation and the critical transition toward chondrocyte maturation and hypertrophy (Bobick and Cobb, 2012).

### 2.3 Skeletal differences between isolated SHOX deficiency and Turner syndrome

Skeletal features in *SHOX* pathologies differ between patients with a normal karyotype (such as isolated LMD) and those with Turner syndrome. Mesomelic features typical of LMD and LWD, which are often milder or absent in Turner syndrome, are primarily caused by *SHOX* dosage effects and the skeletal-maturing effect of gonadal estrogens, which lead to premature growth plate fusion (Ogata et al., 2002; Seki et al., 2014). In Turner syndrome, lymphatic hypoplasia (due to haploinsufficiency of putative lymphogenic genes) is thought to alter skeletal development. Distended cervical lymphatics and facial lymphedema may exert a compressive effect on developing skeletal tissues, contributing to characteristic craniofacial and cervical features, whereas peripheral lymphedema of the distal limbs may facilitate short metacarpals and cubitus valgus (Ogata et al., 2002).

Individuals with isolated *SHOX* deficiency exhibit reduced cortical volumetric bone mineral density and diminished cortical thickness at the radius in heterozygous cohorts (Soucek et al., 2013). Comparable bone-quality studies in LMD are lacking; therefore, these data should not be extrapolated quantitatively to functionally complete SHOX deficiency.

These contrasts between LMD and other SHOX-related conditions are summarised in Table 1, which juxtaposes ISS, LWD, Turner syndrome and LMD across key clinical and molecular features.

**TABLE 1:** Langer mesomelic dysplasia in the context of SHOX-related disorders.

Feature	Idiopathic short stature (ISS)	Léri-Weill dyschondrosteosis (LWD)	Turner syndrome (45,X)	Langer mesomelic dysplasia (LMD)
SHOX status	Heterozygous SHOX deficiency in a subset of patients	Heterozygous null or hypomorphic SHOX allele	SHOX haploinsufficiency due to monosomy X	Biallelic SHOX inactivation or functionally severe biallelic deficiency
OMIM	#300582	#127300	–	#249700
Mode of inheritance	Pseudoautosomal dominant, variable penetrance	Pseudoautosomal dominant	Sporadic chromosomal disorder	Pseudoautosomal recessive
Height deficit	Mild to moderate	Mild to moderate	Moderate	Severe; mean approximately −6.18 SDS calculated from the 10 of 17 cases with numerical SDS values in Fukami et al. (2005) Table 1
Madelung deformity	Absent	Common, particularly in adolescent females	May be present, generally less severe than in LWD	Variable; may be severe, mild, or absent
Mesomelic shortening	Usually absent or subtle	Moderate	Mild or absent	Severe, with hypoplasia/aplasia of the ulna and fibula
Turner-specific somatic features	Absent	Absent	Characteristic, but variable	Absent unless Turner syndrome co-exists
Visceral abnormalities	Absent	Absent	May be present, especially cardiac or renal anomalies	Generally absent
Pathophysiological mechanism	Partial SHOX haploinsufficiency ± other factors	SHOX haploinsufficiency	SHOX haploinsufficiency plus putative lymphogenic-gene effects and estrogen deficiency	Biallelic loss or severe impairment of SHOX function
Representative references	Blaschke and Rappold, 2000; Fukami et al., 2016	Blaschke and Rappold, 2000; Gahunia et al., 2009; Fukami et al., 2016; Vodopiutz et al., 2023	Ogata et al., 2002; Seki et al., 2014	Langer, 1967; Zinn et al., 2002; Fukami et al., 2005; Gahunia et al., 2009; Barca-Tierno et al., 2011

This table contextualises LMD within the broader group of SHOX-related conditions. The mean −6.18 SDS refers to Fukami et al. (2005) Table 1 (calculated from the 10 of 17 cases with numerical SDS values; the remaining seven were fetuses, a neonate, or adults reported without quantitative SDS); later cases are not included in that mean. Reported individual SDS values across the broader LMD literature range from −1.8 to −10.2 SDS. The lower value reflects an unusually mild/complex mosaic 45,X/46,X,r(X) infant, and the upper value is from Barca-Tierno et al. (2011), Family 10 II.7. For clinical orientation, the corresponding adult or near-adult heights in selected affected individuals span approximately 92–120 cm (Barca-Tierno et al., 2011; Kohkalani et al., 2025).

Abbreviations: ISS, idiopathic short stature; LMD, langer mesomelic dysplasia; LWD, Léri-Weill dyschondrosteosis; SHOX, short stature homeobox gene; OMIM, online mendelian inheritance in man; SDS, standard deviation score.

## 3 Clinical features of LMD

### 3.1 Physical examination and radiographic findings

Patients with LMD present with extremely severe short stature (Langer, 1967; Zinn et al., 2002). Based on the 17 cases compiled by Fukami et al. (2005), the mean height deficit was approximately −6.18 standard deviation scores (calculated from the 10 of 17 cases with numerical SDS values; the remaining seven were fetuses, a neonate, or adults reported without quantitative SDS); later published cases are not included in this mean (Fukami et al., 2005; Gahunia et al., 2009). Because the published series combines fetuses, infants, children, and adults, a single mean height expressed in centimeters would be clinically misleading. For orientation, available adult or near-adult reports include absolute heights of approximately 92–120 cm in selected affected individuals, whereas SDS remains the most comparable metric across ages and sexes (Barca-Tierno et al., 2011; Kohkalani et al., 2025). Reported cases of LMD involve various degrees of limb skeletal abnormalities, whereas the cranium, chest, spine, and pelvis typically show normal proportions (Langer, 1967; Gahunia et al., 2009). Although originally considered a defining feature of LMD (Langer, 1967), hypoplastic mandible is now recognized as inconsistently present (Ogata et al., 2002; Fukami et al., 2005).

Radiographic findings demonstrate significant dysplastic and hypoplastic changes in all long bones, with a characteristic and disproportionate mesomelic shortening of the forearms and lower legs. Defective ossification is particularly prominent in the distal ulna and proximal fibula (Langer, 1967; Gahunia et al., 2009).

In the upper limbs, both the radius and ulna exhibit marked deformities; the ulna is typically bowed and hypoplastic, often lacking the distal epiphysis. The radius demonstrates varying degrees of bowing and may be associated with Madelung-type deformity, although this feature shows considerable variability: it may be severe, mild, or even absent in individual patients (Gahunia et al., 2009; Aggarwal et al., 2014; Fukami et al., 2016; Vodopiutz et al., 2023). These mesomelic defects lead to secondary carpal bone distortions and ulnar deviation of the hand (Langer, 1967; Gahunia et al., 2009). Madelung deformity is a characteristic type of forearm defect comprising volar and ulnar tilt of the distal radial articular surface, increased inclination of the distal radial joint surface, triangulation of the carpus, and prominent dorsal subluxation of the ulnar head (Kumar et al., 2013).

In the lower limbs, the tibia and fibula are typically short and dysplastic; however, the relative severity of upper-versus lower-limb involvement varies among published cases (Bieganski et al., 2005; Gahunia et al., 2009). Many patients show severe impairment of both the forearms and lower legs, while some reports describe more conspicuous lower-limb deformity and others emphasize upper-limb Madelung-type changes. The tibia may be short and medially bowed with a broadened diaphysis and an oblique distal articular surface, whereas the fibula is often rudimentary and proximally shortened. The ossification center for the fibular head may be absent, while the tibial epiphyseal ossification centers may appear small and deformed (Kunze and Klemm, 1980), and both bones frequently exhibit varying stages of morphological distortion (Langer, 1967; Kunze and Klemm, 1980; Gahunia et al., 2009). The disproportion between tibial and fibular lengths contributes to characteristic valgus deformities at the knees (genu valgum) and ankles, which may be the presenting feature leading to orthopedic consultation (Bieganski et al., 2005; Aggarwal et al., 2014).

LMD resulting from *SHOX* variants is not associated with visceral abnormalities (Roth et al., 1996; Zinn et al., 2002; Mazza et al., 2011; Ambrosetti et al., 2014). Cognitive development and intellectual potential typically fall within the normal range (Langer, 1967; Zinn et al., 2002; Ambrosetti et al., 2014). While instances of intellectual disability have been reported, these have been attributed either to large deletions encompassing contiguous genes outside PAR1 or, in one published LMD case with a deletion confined to PAR1, to a hypothesized additional recessive locus within PAR1 rather than to *SHOX* nullizygosity itself (Robertson et al., 2000; Zinn et al., 2002).

Conductive hearing loss has been documented in patients with LMD, notably in a Turkish cohort where two of four patients with LMD showed bilateral conductive hearing loss (45–65 dB) (Gürsoy et al., 2020). Whether this represents a rare phenotypic manifestation of SHOX deficiency or a coincidental finding in a consanguineous family remains unclear; systematic audiological evaluation in larger cohorts will be essential to determine the true prevalence.

Recurrent dermatoglyphic abnormalities were not described in the reviewed LMD reports. The statement that the cranium is usually proportionate is based on conventional clinical and radiographic descriptions; CT-based morphometric studies or 3D craniofacial models comparing head shape with normative population data were not identified. A severe, atypical presentation of dyschondrosteosis has been described in a mother and son with a “hybrid” phenotype: their upper extremities exhibited characteristic features of LWD, while their lower extremities were more consistent with LMD. Molecular analysis revealed only a heterozygous *SHOX* deletion in both patients, indicating that severe LMD-like skeletal features can arise from *SHOX* haploinsufficiency alone (Bieganski et al., 2005).

Table 2 organises the characteristic clinical and radiographic findings of LMD by anatomical region, with corresponding source references.

**TABLE 2:** Characteristic clinical and radiographic features of LMD organised by anatomical region.

Anatomical region	Characteristic clinical and radiographic findings	References
Cranium and mandible	Cranium typically of normal proportions in conventional clinical/radiographic descriptions. Mandibular hypoplasia/micrognathia is inconsistently present. No CT-based morphometric or 3D head-model comparison with normative craniofacial data was identified in the reviewed LMD literature	Langer, 1967; Ogata et al., 2002; Fukami et al., 2005; Bertorelli et al., 2007; Ambrosetti et al., 2014
Thorax, spine and pelvis	Generally proportionate in classical isolated LMD, without major structural abnormalities	Langer, 1967; Gahunia et al., 2009
Stature	Severe short stature; mean approximately −6.18 SDS calculated from the 10 of 17 cases with numerical SDS values in Fukami et al. (2005) Table 1 (the remaining seven were fetuses, a neonate, or adults with no quantitative SDS reported), with broader reported values from −1.8 to −10.2 SDS. Because reported patients span fetal to adult ages, a single mean height in centimeters is not clinically robust; selected adult or near-adult reports include heights of approximately 92–120 cm	Fukami et al., 2005; Gahunia et al., 2009; Barca-Tierno et al., 2011
Ulna	Bowed and hypoplastic, often lacking the distal epiphysis; defective ossification especially in the distal ulna	Langer, 1967; Kunze and Klemm, 1980; Gahunia et al., 2009
Radius	Variable bowing; Madelung-type deformity may be present but varies from severe to mild or absent	Aggarwal et al., 2014; Fukami et al., 2016; Vodopiutz et al., 2023; Gahunia et al., 2009
Wrist and hand	Ulnar deviation and secondary carpal distortion may occur; hands are relatively preserved. When present, Madelung-type deformity includes distal radial tilt, carpal triangulation, and dorsal ulnar subluxation	Langer, 1967; Gahunia et al., 2009; Kumar et al., 2013
Tibia	Short and medially bowed with broadened diaphysis and oblique distal articular surface; epiphyseal centres may be small and deformed	Langer, 1967; Kunze and Klemm, 1980; Gahunia et al., 2009
Fibula	Often rudimentary and proximally shortened; fibular-head ossification centre may be absent	Langer, 1967; Kunze and Klemm, 1980; Gahunia et al., 2009
Knees and ankles	Tibia-fibula disproportion may produce genu valgum and ankle valgus; these deformities can prompt orthopaedic consultation	Bieganski et al., 2005; Aggarwal et al., 2014
Hands and feet	Relatively normal size; digits typically spared; no characteristic nail abnormalities	Langer, 1967; Zinn et al., 2002
Visceral involvement	Generally absent in classical isolated LMD; additional non-skeletal findings suggest contiguous-gene effects or an alternative/overlapping diagnosis	Roth et al., 1996; Zinn et al., 2002; Mazza et al., 2011; Ambrosetti et al., 2014

Abbreviations: LMD, langer mesomelic dysplasia; SDS, standard deviation score.

### 3.2 Prenatal findings

Features of LMD can be identified via prenatal ultrasonography, particularly in families with a known history of mesomelic dysplasia. Any clinical suspicion of LMD should be validated through molecular genetic testing and specialized genetic counseling for the family. While the earliest documented sonographic suspicion of LMD occurred at 12 + 3 weeks of gestation, most prenatally identified cases resulted in second-trimester termination of pregnancy, with the diagnosis subsequently confirmed by genetic testing. When familial variants are known or when fetal imaging strongly suggests LMD, SHOX status can be assessed using DNA obtained through invasive prenatal diagnostic procedures, including chorionic villus sampling, amniocentesis, or cordocentesis when clinically indicated (Mazza et al., 2011; Ambrosetti et al., 2014).

Characteristic findings include significant shortening of the upper and lower limbs (typically falling below the fifth percentile on fetal growth curves (Mazza et al., 2011; Ambrosetti et al., 2014)), with the mesomelic segments most severely affected. These deformities, primarily involving the radius, ulna, tibia, and fibula, range from bone shortening and bowing to complete aplasia. Hands and feet are generally of normal morphology, and LMD is usually isolated to the skeleton (Roth et al., 1996; Thomas et al., 2004).

The histopathological characterization of LMD tissue architecture has been primarily documented using fetal bone specimens obtained following the medical termination of pregnancy. In the growth plate of LMD fetuses, the mutant SHOX protein has been detected across the reserve, proliferative, and hypertrophic zones. However, significant cellular disorganization was evident in the proliferative zone, where chondrocyte columns were shortened and stacked side-by-side rather than in a normal longitudinal orientation. Additionally, enlargement of chondrocytes was observed in the reserve zone. These findings support a role for *SHOX* in chondrocyte column organization and growth plate architecture (Thomas et al., 2004; Barca-Tierno et al., 2011).

### 3.3 Obstetric issues in women with *SHOX*-related mesomelic dysplasia

Evidence regarding pregnancy and delivery in women with LMD or other *SHOX*-related mesomelic dysplasias remains limited. Existing reports describe successful vaginal delivery, and the mode of delivery should be individualized by obstetric assessment rather than determined solely by the *SHOX*-related diagnosis. Although the trunk and pelvis may maintain relatively normal proportions in LMD, pelvic dimensions, fetal presentation, fetal size, maternal comorbidities, and standard obstetric indications should guide the final decision. Anaesthetic and monitoring issues may require special consideration because upper-limb deformities can alter or preclude conventional blood pressure monitoring. Mesomelic shortening can make the auscultation of Korotkoff sounds and the assessment of peripheral pulses difficult or impossible. In such cases, non-invasive continuous arterial pressure monitoring via the finger has been employed successfully (Clark et al., 1993).

### 3.4 Growth hormone therapy in LMD

Evidence regarding the efficacy of growth hormone (GH) therapy in LMD remains limited. *SHOX* haploinsufficiency typically results in significant short stature without spontaneous catch-up growth, as observed in LWD, Turner syndrome, and *SHOX*-related ISS. While GH supplementation has demonstrated therapeutic benefits in Turner syndrome and other *SHOX*-haploinsufficient conditions, often inducing sustained catch-up growth at standard dosages, its efficacy in LMD is less certain (Blum et al., 2007; Shah et al., 2009; Fukami et al., 2016).

Randomized controlled trials involving patients with *SHOX* haploinsufficiency have demonstrated a statistically significant response to GH therapy, characterized by marked improvements in height velocity and height standard deviation scores over a 2-year period (Blum et al., 2007). However, it remains uncertain whether these findings can be extrapolated to cases of complete functional *SHOX* nullity, as observed in LMD. In particular, GH therapy failed to produce growth acceleration in a documented case involving a combination of LMD and Turner syndrome. Despite a biochemical response characterized by rising IGF-1 levels, there was no corresponding clinical improvement in height velocity, and the patient’s height deficit increased over 4 years of treatment. The authors concluded that GH therapy may not be beneficial in the setting of complete SHOX deficiency, although no worsening of the skeletal deformities was observed (Shah et al., 2009).

## 4 Genetics of LMD: *SHOX* variants

Although *SHOX* deficiency has high penetrance, its clinical expressivity is remarkably variable, demonstrating a weak genotype-phenotype correlation. Phenotypic diversity is frequently observed even among family members carrying the same genetic alteration (Ogata et al., 2002; Alvarez-Mora et al., 2012). Current evidence suggests that the type of variant, including the extent of deletions or the presence of sequence variants, does not reliably predict the severity of the resulting disease (Alvarez-Mora et al., 2012; Poggi et al., 2015).

Table 3 compiles the reported molecularly characterised LMD cases discussed in the following subsections, organised by case index with paternal and maternal alleles, the inferred molecular mechanism, and the original reference.

**TABLE 3:** Reported cases of LMD with documented or pedigree-inferred SHOX genotype.

Case	Age/sex	Height (SDS)	Paternal allele/Father	Maternal allele/Mother	Molecular mechanism	References
1	Female fetus (24 weeks; 45,X)	n.r	Paternal X absent	SHOX deletion (LWD mother)	Functional SHOX nullizygosity: 45,X plus maternal X bearing SHOX deletion	Belin et al., 1998
2	Female fetus (20 weeks)	n.r	PAR1 deletion close to/within SHOX (father with short stature, no Madelung)	PAR1 deletion encompassing SHOX (mother with short stature, no mesomelia/Madelung)	Biallelic PAR1 deletions involving SHOX	Shears et al., 1998
3	12 years, male (MR, seizures)	−7.2	SHOX deletion in PAR1 (LWD father)	SHOX deletion in PAR1 (LWD mother)	Homozygous SHOX deletion confined to PAR1; MR/seizures hypothesized by Robertson et al. (2000) to reflect a putative recessive MRX/Y locus within PAR1 rather than SHOX nullizygosity	Robertson et al., 2000
4	85 years, female (Zinn subject SW464)	−5.5	R153C; parental origin not determined	V163F; parental origin not determined	Compound heterozygous homeodomain missense variants in trans	Zinn et al., 2002
5	7 years, female (Zinn subject SW479)	−5.5	ins350AG/R118fsX130 (father with borderline short stature)	Complete SHOX deletion (LWD mother)	Complete SHOX deletion + truncating frameshift	Zinn et al., 2002; Fukami et al., 2005
6	65 years, male (Zinn subject SW474)	−7.2	ins723C/P243fsX321; parental origin not determined	Second allele not characterized; partial exon 6a deletion not excluded	Hemizygous or homozygous exon 6a frameshift (ins723C/P243fsX321); small intragenic deletion of the second allele could not be excluded	Zinn et al., 2002; Fukami et al., 2005
7	24 years, female (pedigree V:3)	−8.9	R173C (LMD-like father, not molecularly tested)	R173C (LWD mother)	Homozygous R173C missense at NLS; pseudo-dominant inheritance	Shears et al., 2002
8	Newborn male (pedigree VI:1)	n.r	R173C (LWD father)	R173C (LMD mother, case 7)	Homozygous R173C missense at NLS	Shears et al., 2002
9	1.5 years, male (Japanese)	−4.2	SHOX deletion (LWD father)	R168W missense (LWD mother)	SHOX deletion + homeodomain missense	Ogata et al., 2002
10	Female fetus (20 weeks)	n.r	*De novo* del(X)(p22.12) ∼16 Mb including SHOX	SHOX microdeletion ∼170–250 kb	Paternal *de novo* cytogenetic deletion + maternal submicroscopic deletion	Thomas et al., 2004
11	Adult, male (pedigree IV.2; Spanish Romani)	SS (no SDS)	A170P (LWD father)	A170P (LWD mother)	Homozygous A170P at NLS, molecularly confirmed; founder variant	Barca-Tierno et al., 2011; Sabherwal et al., 2004a
12^a^	38 years, female (pedigree IV.5; Sabherwal IV-4)	−9.1^b^	Presumed A170P from LWD carrier father	Presumed A170P from LWD carrier mother	Presumed homozygous A170P; clinical Langer dysplasia/LMD, not directly molecularly tested in Sabherwal et al. (2004a) or Barca-Tierno et al. (2011)	Barca-Tierno et al., 2011; Sabherwal et al., 2004a
13^a^	Adult, female (pedigree IV.8; Sabherwal IV-7)	−9.0	Presumed A170P from LWD carrier father	Presumed A170P from LWD carrier mother	Presumed homozygous A170P; clinical Langer dysplasia/LMD, not directly molecularly tested in Sabherwal et al. (2004a) or Barca-Tierno et al. (2011)	Barca-Tierno et al., 2011; Sabherwal et al., 2004a
14	7 years, female (pedigree V.9; Sabherwal V-6)	−8.0	A170P (LWD father)	A170P (presumed LMD mother, case 13)	Homozygous A170P at NLS, molecularly confirmed in Sabherwal et al. (2004a)	Barca-Tierno et al., 2011; Sabherwal et al., 2004a
15	35 years, female (Zinn subject SW406)	−6.8	Complete SHOX deletion (parental origin not determined; both parents LWD)	Second allele uncharacterized (parental origin not determined; both parents LWD)	SHOX deletion + uncharacterized second allele; sibling of case 16	Zinn et al., 2002
16	33 years, male (Zinn subject SW442)	−6.2	Complete SHOX deletion (parental origin not determined; both parents LWD)	Second allele uncharacterized (parental origin not determined; both parents LWD)	SHOX deletion + uncharacterized second allele; sibling of case 15	Zinn et al., 2002
17	1.5 years, female (mosaic 45,X/46,X,r(X))	−1.8	SHOX absent from paternal ring X	240–350 kb 3′ enhancer-region deletion	Paternal ring X SHOX loss + maternal enhancer deletion; mild/complex LMD-compatible presentation	Fukami et al., 2005
18	22-week male fetus (46,XY)	n.r	1.1 Mb downstream enhancer deletion (LWD father)	1.1 Mb downstream enhancer deletion (LWD mother)	Homozygous downstream enhancer deletion; consanguineous parents	Bertorelli et al., 2007; Ambrosetti et al., 2014
19	12 + 3 weeks female fetus	n.r	1.1 Mb downstream enhancer deletion (LWD father)	1.1 Mb downstream enhancer deletion (LWD mother)	Homozygous downstream enhancer deletion; same parental couple/family as case 18	Mazza et al., 2011; Ambrosetti et al., 2014
20	50 years, male (pedigree III.3)	−4.16	SHOX-encompassing PAR1 deletion 106–255 kb (LWD father)	Downstream PAR1 deletion 199–319 kb, no SHOX (LWD mother)	SHOX-encompassing deletion + downstream enhancer deletion	Campos-Barros et al., 2007
21	21 months, male (Japanese IVF dizygotic twin)	−3.9 at 21 months; −1.1 SD at birth	∼500 kb Y deletion ∼300 kb downstream of SHOX (clinically normal father with normal stature)	∼46 kb X deletion including SHOX exons 1–5 (mother with mesomelic short stature and limited wrist mobility)	Maternal coding deletion + paternal downstream deletion; mild LMD phenotype	Tsuchiya et al., 2014
22	4 siblings (Turkish family)	Median −5.6 (range −7.65 to −4.65)	c.42delG frameshift (LWD father)	c.42delG frameshift (LWD mother)	Homozygous p.Ser16AlafsTer60 frameshift; 2/4 with bilateral conductive hearing loss	Gürsoy et al., 2020
23	30 years, male (IV-2) + 27 years, female (IV-4)	SDS n.r.; 113.5/92 cm	c.486 + 2T>C splice-donor; ISS carrier parents/relatives	c.486 + 2T>C splice-donor; ISS carrier parents/relatives	Homozygous splice-donor variant in two related LMD individuals from different sibships	Kohkalani et al., 2025
24	LMD patient (Bunyan patient 375)	n.r	Whole SHOX gene deletion	Whole SHOX gene deletion	Homozygous whole SHOX deletion	Bunyan et al., 2013
25	LMD patient (Bunyan patient 220)	n.r	249 kb deletion 3′ of SHOX	249 kb deletion 3′ of SHOX	Homozygous downstream enhancer deletion	Bunyan et al., 2013
26	Adult, female (family 10, pedigree II.7)	−10.2	A170P; parental phenotype not reported	A170P; parental phenotype not reported	Homozygous A170P, genetically confirmed; Madelung deformity present; most extreme reported LMD short stature	Barca-Tierno et al., 2011
27	22-week male fetus (family 11, pedigree IV.9)	n.r	A170P (LWD father)	A170P (LWD mother)	Homozygous A170P; genetically confirmed prenatal diagnosis	Barca-Tierno et al., 2011
28	18-week female fetus (family 2, pedigree IV.4)	n.r	A170P (LWD father)	A170P (LWD mother)	Homozygous A170P; genetically confirmed prenatal diagnosis	Barca-Tierno et al., 2011
29^a^	Adult, male (family 9, pedigree I.1)	−8.2	Presumed A170P; parental phenotype not reported	Presumed A170P; parental phenotype not reported	Presumed homozygous A170P; clinical Langer dysplasia (height 120 cm, Madelung deformity present), not directly molecularly tested in Barca-Tierno et al. (2011)	Barca-Tierno et al., 2011
30^a^	Female fetus (family 2, pedigree IV.1)	n.r	Presumed A170P (LWD father III.1)	Presumed A170P (LWD mother III.2)	Presumed homozygous A170P; clinical Langer dysplasia (short stature, no Madelung), not directly molecularly tested in Barca-Tierno et al. (2011)	Barca-Tierno et al., 2011
31^a^	Child, male (family 2, pedigree IV.7)	n.r	Presumed A170P (LWD father III.8)	Presumed A170P (LWD mother III.7)	Presumed homozygous A170P; clinical Langer dysplasia (short stature, no Madelung), not directly molecularly tested in Barca-Tierno et al. (2011)	Barca-Tierno et al., 2011

The table includes only individuals classified as LMD, defined here as biallelic or functionally biallelic SHOX involvement, either directly documented or inferred from segregation within molecularly characterised families. Cases 1–17 are based on the Fukami et al. (2005) compilation, whereas cases 18–31 represent subsequently published cases. LWD-only relatives were not included. Original subject and pedigree identifiers were retained to allow cross-checking with the source reports; pedigree identifier formatting (e.g., IV.2, V-6, V:3) follows the conventions of the original publications.

Variant nomenclature is retained as in the original publications. cDNA, and protein numbering follows the *SHOXa* transcript (RefSeq NM_000451.4 / NP_000442.1); genomic coordinates reported in earlier articles may correspond to earlier genome builds.

^a^Cases 12, 13 and 29–31 were not directly molecularly confirmed in the original reports but were retained because they were clinically diagnosed as LMD, within molecularly characterised A170P families.

^b^For case 12, the table uses the later Barca-Tierno et al. SDS value.

Abbreviations: LMD, langer mesomelic dysplasia; LWD, Léri-Weill dyschondrosteosis; SHOX, short stature homeobox gene; PAR1, pseudoautosomal region 1; ISS, idiopathic short stature; SDS, standard deviation score; SD, standard deviation; MR, mental retardation; MRX/Y, X- and Y-linked mental retardation locus; NLS, nuclear localization signal; IVF, *in vitro* fertilisation; n.r., not reported; SS, short stature reported without quantitative SDS; wk, weeks; mo, months.

To address the clinical variability across published cases, Table 4 maps the same case indices used in Table 3 to the key clinical and radiographic findings reported in the source publications. Where individualized descriptions were not provided in the primary report or subsequent review, this is stated explicitly rather than inferred.

**TABLE 4:** Clinical and radiographic findings in reported LMD patients corresponding to the molecularly characterized cases in Table 3.

Case(s) in Table 3	Source/age	Key clinical findings	Key radiographic or limb-pattern findings
1	Female fetus, 24 weeks; Belin et al., 1998	Prenatal LMD in a 45,X fetus with maternal SHOX deletion; no postnatal clinical course	Severe mesomelic skeletal dysplasia; individualized segment-level radiographic details not reported in detail
2	Female fetus, 20 weeks; Shears et al., 1998	Fetal mesomelic dwarfism in a family with parental short stature	Severe limb shortening attributed to biallelic PAR1/SHOX deletions; individualized radiographic details limited
3	12-year male; Robertson et al., 2000	Severe short stature with LMD; intellectual disability and seizures were considered unlikely to result from SHOX nullizygosity alone	Classical LMD skeletal pattern with severe mesomelia; detailed regional radiographic description limited in the review sources
4	85-year female; Zinn et al., 2002	Adult LMD with compound homeodomain missense variants and severe short stature; high-arched palate, short fourth metacarpal and marked increased carrying angle were reported	Severe mesomelic shortening consistent with complete functional SHOX deficiency; individualized regional radiographic description in the source was limited
5	7-year female; Zinn et al., 2002	Childhood LMD due to complete deletion plus frameshift variant; high-arched palate and marked increased carrying angle were reported	Source radiographs showed radial bowing, severe mesomelia, Madelung deformity, ulnar hypoplasia, hypoplastic fibulae and tibial bowing
6	65-year male; Zinn et al., 2002	Adult LMD with frameshift variant and an uncharacterized second allele (partial exon 6a deletion not excluded); high-arched palate and mild increased carrying angle were reported	Severe mesomelic dysplasia; individualized regional radiographic illustration/details limited in the source
7	24-year female; Shears et al., 2002	Pseudodominant familial LMD; severe short stature	Homozygous homeodomain/NLS missense variant with classical LMD-type limb disproportion
8	Newborn male; Shears et al., 2002	Newborn affected in the same R173C family	Classical LMD-type skeletal phenotype; individualized details n.r
9	1.5-year male; Ogata et al., 2002	Japanese child with SHOX nullizygosity and severe disproportionate short stature	LMD-compatible mesomelic shortening; SHOX-related Turner-like skeletal features discussed in the source
10	Female fetus, 20 weeks; Thomas et al., 2004	Prenatal LMD after fetal skeletal abnormality and large paternal X-chromosome deletion plus maternal SHOX microdeletion	Severe fetal mesomelic limb shortening; diagnosis confirmed after molecular testing
11	Adult male; Sabherwal et al., 2004a/Barca-Tierno et al., 2011	Spanish Romani family with A170P; adult male with clinical Langer dysplasia	Madelung deformity/mesomelic dysplasia within homozygous A170P family; individual detail limited
12	38-year female; Sabherwal et al., 2004a/Barca-Tierno et al., 2011	Clinical Langer dysplasia/LMD in an A170P family; severe adult short stature	Mesomelic dysplasia; individual molecular confirmation not available in the original reports
13	Adult female; Sabherwal et al., 2004a/Barca-Tierno et al., 2011	Clinical Langer dysplasia/LMD in an A170P family	Mesomelic dysplasia; individual molecular confirmation not available in the original reports
14	7-year female; Sabherwal et al., 2004a/Barca-Tierno et al., 2011	Molecularly confirmed homozygous A170P in childhood	LMD-type mesomelic limb shortening in the A170P family
15	35-year female; Zinn et al., 2002	Adult LMD sibling pair with SHOX deletion plus uncharacterized second allele; high-arched palate and mild increased carrying angle were reported	Severe mesomelic dysplasia; individualized regional radiographic illustration/details limited in the source
16	33-year male; Zinn et al., 2002	Adult LMD sibling pair with SHOX deletion plus uncharacterized second allele; high-arched palate, short fourth metacarpal and mild increased carrying angle were reported	Source radiographs showed radial bowing, severe mesomelia, Madelung deformity, ulnar hypoplasia, hypoplastic fibulae and tibial bowing
17	1.5-year female; Fukami et al., 2005	Mild/complex LMD-compatible phenotype in mosaic 45,X/46,X,r(X) infant	Relatively mild skeletal phenotype compared with classic LMD; distal SHOX enhancer deletion plus ring X lacking SHOX.
18	Male fetus, 22 weeks; Bertorelli et al., 2007/Ambrosetti et al., 2014	Fetus with disproportionate mesomelic shortening, micrognathia, normal visceral findings	Severe ulnar and fibular hypoplasia with bowed and moderately hypoplastic radii and tibiae; ribs, vertebrae and pelvic bones described as normal
19	Female fetus, 12 + 3 weeks; Mazza et al., 2011/Ambrosetti et al., 2014	Earliest reported first-trimester ultrasonographic suspicion in an affected family; normal visceral organs	Mesomelic shortening and bowing of upper and lower limbs; ulnar deviation; unossified radii, ulnae, fibulae and tibiae at this gestational age
20	50-year male; Campos-Barros et al., 2007	Adult LMD caused by a SHOX-encompassing deletion in trans with a downstream PAR1 deletion; final height 146.7 cm (−4.16 SD), high-arched palate, Madelung deformity and severe increased carrying angle	Very short, bowed forearms and lower legs; marked rhizomelic/mesomelic upper-limb shortening; the fibula was described as normal/not proximally hypoplastic, making this case milder or atypical for classic LMD.
21	21-month male; Tsuchiya et al., 2014	Mild LMD presentation in an IVF dizygotic twin; short extremities were detected prenatally, and at 21 months height was 69.3 cm (−3.9 SD) with obvious mesomelic short stature	Bone survey showed markedly curved radii, hypoplastic ulnae and fibulae, and metaphyseal splaying; skeletal findings were milder than in classical LMD.
22	Four siblings; Gürsoy et al., 2020	Four Turkish siblings with homozygous c.42delG frameshift and disproportionate short stature; short/webbed neck, low hairline, pectus excavatum, severe Madelung deformity with ulnar deviation, camptodactyly, cubitus valgus and muscular hypertrophy were reported; two had bilateral conductive hearing loss (45–65 dB)	Radiographs showed bowing and shortening of the distal radius, widening of the distal radioulnar joint, and triangularization of the distal radial epiphysis with ulnar slant; ulnar deviation and camptodactyly were also reported clinically
23	Two related adults, male and female; Kohkalani et al., 2025	Two related adults with homozygous c.486 + 2T>C splice-donor variant; source reported heights of 113.5 and 92 cm, Madelung deformity and genu varum	Madelung deformity and genu varum were recorded; detailed segment-level radiographic description was not provided
24	Bunyan patient 375; Bunyan et al., 2013	LMD patient detected during diagnostic screening	Homozygous whole-SHOX deletion; individualized clinical/radiographic detail n.r
25	Bunyan patient 220; Bunyan et al., 2013	LMD patient detected during diagnostic screening	Homozygous downstream enhancer deletion; individualized clinical/radiographic detail n.r
26	Adult female, family 10 II.7; Barca-Tierno et al., 2011	Most extreme reported short stature in Table 3 (−10.2 SDS); Madelung deformity present	Homozygous A170P with severe LMD skeletal phenotype
27	Male fetus, 22 weeks, family 11 IV.9; Barca-Tierno et al., 2011	Prenatal diagnosis in an A170P family	Homozygous A170P with fetal LMD phenotype; detailed radiographic description n.r
28	Female fetus, 18 weeks, family 2 IV.4; Barca-Tierno et al., 2011	Prenatal diagnosis in an A170P family	Homozygous A170P with fetal LMD phenotype; detailed radiographic description n.r
29	Adult male, family 9 I.1; Barca-Tierno et al., 2011	Clinical Langer dysplasia with height around 120 cm and Madelung deformity present	Presumed homozygous A170P; radiographic detail limited in source summary
30	Female fetus, family 2 IV.1; Barca-Tierno et al., 2011	Clinical Langer dysplasia; short stature; Madelung deformity not reported	Presumed homozygous A170P; detailed radiographic description n.r
31	Child male, family 2 IV.7; Barca-Tierno et al., 2011	Clinical Langer dysplasia; short stature; Madelung deformity not reported	Presumed homozygous A170P; detailed radiographic description n.r

This table emphasizes clinically useful phenotypic and radiographic details.

Abbreviations: n.r., not reported in detail in the available individualized source description; LMD, langer mesomelic dysplasia; LWD, Léri-Weill dyschondrosteosis; SDS, standard deviation score.

### 4.1 Deletions

Observations of LMD-affected offspring born to two parents with LWD led to the hypothesis that LMD represents the homozygous (or compound heterozygous) manifestation of *SHOX* deficiency. The clinical expression of such variants in heterozygotes is highly variable, ranging from subclinical forearm anomalies to classic LWD. In many instances, heterozygous carriers present with subtle skeletal changes that may remain undetected until identified through pedigree analysis or molecular screening during genetic counseling (Kunze and Klemm, 1980; Goldblatt et al., 1987; Beighton, 1997).

The observation of Madelung deformity in the parents of children with LMD initially suggested that the disorder resulted from homozygous variants. Linkage analysis of families with LWD localized the causative gene to the DXYS233 locus in the pseudoautosomal region, where the *SHOX* gene had previously been mapped. These studies ultimately established that the complete loss of functional *SHOX*, or a functional null state, is the underlying cause of LMD (Belin et al., 1998; Shears et al., 1998; Cormier-Daire et al., 1999; Zinn et al., 2002).

The first molecular evidence for *SHOX* nullizygosity as the underlying etiology of LMD was established through genetic investigations of affected fetuses. Belin et al. reported a 24-week fetus with the radiographic appearance of LMD and a 45,X karyotype; the single X chromosome, inherited from the mother, carried a *SHOX*-encompassing pseudoautosomal microdeletion, rendering the fetus nullizygous for *SHOX* (Belin et al., 1998). Shears et al. described a 20-week fetus with LMD who was a compound heterozygote, carrying *SHOX* deletions inherited from both parents (Shears et al., 1998). A third case involved a 20-week fetus who carried an inherited maternal *SHOX* microdeletion alongside a *de novo* paternal Xp terminal deletion also encompassing the *SHOX* locus (Thomas et al., 2004).

Subsequently, the first confirmation of complete *SHOX* deficiency in living patients with classic LMD was reported in a study of five postnatal individuals. *SHOX* abnormalities were detected in all probands, including homozygous or compound heterozygous variants. In the three unrelated cases, pathogenic variants in both alleles were demonstrated, definitively establishing that LMD results from biallelic loss of *SHOX* function. The parents of affected individuals displayed clinical features consistent with LWD (Zinn et al., 2002).

Intron 3 of the *SHOX* gene represents a significant hotspot for partial gene rearrangements; in their cohort, Benito-Sanz et al. (2017) reported breakpoints within intron 3 in 12 of 28 (42.8%) partial deletions and duplications, and a broader review of the literature identified breakpoints in intron 3 in 13 of 21 (62%) partial deletions and 23 of 38 (60.5%) partial duplications. Multiple molecular mechanisms, including non-allelic homologous recombination involving Alu repeat elements, have been implicated in the genesis of these alterations (Benito-Sanz et al., 2017).

A specific 47.5 kb deletion located 160 kb downstream of the *SHOX* gene was identified as the most common downstream deletion in a large screening study. Three probands were compound heterozygous for this deletion and a previously identified pathogenic *SHOX* variant. Although their clinical presentations were largely consistent with LWD rather than LMD, the variable phenotypic expression, ranging from apparently unaffected to classic LWD, highlights the significant phenotypic heterogeneity associated with *SHOX* variants (Bunyan et al., 2013).

### 4.2 Sequence variants

As with other skeletal dysplasias, parent-to-child transmission of LMD can occur, typically through a pseudodominant inheritance pattern in consanguineous families. One documented case involved a C517T transition in the *SHOX* homeobox, resulting in a p.Arg173Cys (R173C) missense variant. This substitution is located within the highly conserved recognition helix of the homeodomain, where it is predicted to destabilize the interaction between the SHOX protein and its target DNA sequences (Shears et al., 2002). This case represents the first documented vertical transmission of LMD; transmission across four generations of the kindred was made possible by repeated consanguineous unions linking LWD heterozygotes with LMD homozygotes.

Similar *SHOX* missense variants (p.Ala170Pro [c.508G>C] and p.Ala170Asp [c.509C>A]) have been identified in Spanish families. The A170P substitution segregates with LWD in heterozygotes and with LMD in homozygotes, while A170D has so far been described only in heterozygous LWD families. Both variants affect the same alanine residue at position 170 within the homeodomain, which forms part of the *SHOX* nuclear localization signal; functional studies demonstrated that both variants impair nuclear translocation of the SHOX protein (Barca-Tierno et al., 2011). In one large consanguineous Spanish Romani kindred segregating for A170P, an unusual father-to-son transmission was documented: a pseudoautosomal crossover during paternal meiosis transferred the originally X-chromosomal variant onto the Y chromosome, and consanguineous parental marriage resulted in homozygosity of A170P on both sex chromosomes of the affected son (Sabherwal et al., 2004a).

These variants could disrupt the nuclear localization signal (NLS), thereby impairing the nuclear translocation of the SHOX protein and resulting in a loss of transcriptional activity. For R173C, the loss of a basic arginine residue and its replacement with an uncharged cysteine appears to interfere with the charge-dependent recognition of the NLS, while the A170P substitution is thought to disrupt the structure of the homeodomain recognition helix through proline-induced backbone bending rather than through direct charge effects (Sabherwal et al., 2004a). In homozygous individuals, these structural defects prevent the SHOX protein from entering the nucleus, thereby manifesting as LMD. This mechanism of functional nullity produces a clinical severity comparable to that observed in patients with complete biallelic *SHOX* locus deletions (Zinn et al., 2002; Sabherwal et al., 2004b).

### 4.3 Enhancer-region variants

Historically, conventional molecular techniques, such as Sanger sequencing and targeted deletion analysis, failed to identify pathogenic variants in a subset of families presenting with LMD. The implementation of multiplex ligation-dependent probe amplification (MLPA) and array comparative genomic hybridization (array-CGH) enabled higher-resolution assessment of the *SHOX* genomic landscape, including its distal regulatory elements (Benito-Sanz et al., 2017). The first description of a homozygous deletion of the *SHOX* 3′ enhancer region causing LMD confirmed enhancer silencing as a mechanism underlying *SHOX*-related skeletal dysplasias (Bertorelli et al., 2007).

Another case involved an infant with skeletal abnormalities consistent with LMD despite no detectable pathogenic variant in the *SHOX* coding sequence. Further investigation revealed mosaicism for a paternally derived ring X chromosome lacking the *SHOX* locus, while the cytogenetically normal X chromosome harbored a microdeletion in the distal 3′ *SHOX* enhancer region. This case, preceding the description by Bertorelli et al. (2007) of pure homozygous enhancer deletion, demonstrated the pathogenic potential of *SHOX* regulatory element loss in the absence of coding-sequence variants (Fukami et al., 2005).

In another instance, an individual with LMD was identified as a compound heterozygote for a *SHOX* gene deletion and a downstream PAR1 deletion. These variants occurred in a trans configuration, where one allele lacked the *SHOX* coding sequence and the homologous allele lacked the critical distal regulatory enhancers within PAR1. This genetic configuration effectively resulted in a functional null state, demonstrating that the loss of regulatory elements on one allele combined with a coding-region deletion on the homologous allele is sufficient to cause LMD (Campos-Barros et al., 2007).

Because available cases are few, genotype-phenotype correlations in patients with downstream *SHOX*-related LMD remain difficult to establish. Rare reports show that LMD can result from an inherited PAR1 deletion combined with a sex-chromosome rearrangement or loss affecting the homologous allele, including paternal ring X, maternal *SHOX* deletions with loss of the paternal X chromosome, and *de novo* paternal X-chromosome deletions (Belin et al., 1998; Thomas et al., 2004; Fukami et al., 2005; Kant et al., 2011).

Combinations of heterozygous deletions within the PAR1 region can occasionally manifest as an intermediate phenotype, characterized by skeletal malformations more suggestive of LMD than LWD. In a reported case of a male infant, a deletion on the X chromosome resulted in a complete loss of *SHOX* function, while a concurrent deletion on the Y chromosome did not involve the coding exons or established enhancer regions. One hypothesis to explain this severity suggests the existence of unidentified *cis*-regulatory elements, implying that coordinated action between multiple enhancers is essential for adequate *SHOX* expression. Alternatively, the Y-chromosome deletion may represent a neutral copy-number variant; this is supported by the infant’s father, who carried the same Y deletion but remained asymptomatic. Consequently, the infant’s presentation may represent the extreme end of the LWD phenotypic spectrum (Tsuchiya et al., 2014).

The *SHOX* gene is subject to a high rate of meiotic recombination between the X and Y chromosomes within the PAR1 locus. Consequently, a variant originally located on the paternal Y chromosome may be transferred to the X chromosome, or *vice versa*, during spermatogenesis. This genomic instability necessitates careful genetic counseling for affected males, as inheritance can follow pseudoautosomal dominant rules despite localization on the sex chromosomes (Kant et al., 2011; Fukami et al., 2016).

### 4.4 Splice-site variants

Recently, a novel splice-donor site variant in the *SHOX* gene, c.486 + 2T>C, was identified as a pathogenic cause of LMD in the homozygous state in an Iranian family. The same variant in the heterozygous state segregated with idiopathic short stature (ISS) in five family members, providing a strong genotype-phenotype correlation consistent with the dose-dependent nature of *SHOX* function. This substitution occurs within the highly conserved +2 position of the 5′ splice site, a region essential for U1 small nuclear RNA recognition. The variant is predicted to disrupt canonical splicing, likely causing severe reduction or loss of functional SHOX protein and the severe skeletal phenotype characteristic of LMD (Kohkalani et al., 2025).

A homozygous leaky splice-site variant in SHOX intron 3 (c.544 + 5G>C) was recently identified as a cause of LWD. This variant permits residual normal splicing while also generating aberrant transcripts predicted to undergo nonsense-mediated mRNA decay, resulting in a biallelic hypomorphic state and overall SHOX haploinsufficiency (Vodopiutz et al., 2023). This observation supports a dosage-based model of SHOX-related phenotypes, in which residual SHOX activity may modulate disease severity. In the context of LMD, compound heterozygosity involving a null allele and a hypomorphic allele could therefore plausibly produce an intermediate phenotype between LWD and classic LMD (Tsuchiya et al., 2014).

Table 5 organises the molecular categories of *SHOX* variants causing LMD discussed in Sections 4.1–4.4, with representative variants and key references.

**TABLE 5:** Molecular classification of *SHOX* variants causing LMD.

Variant type	Manuscript section	Molecular mechanism	Representative variants	References	Suitable laboratory methods
Whole or partial SHOX deletions	4.1	Loss of all or part of the SHOX coding sequence; recurrent breakpoints, including an intron 3 hotspot	Complete SHOX deletions; partial intragenic deletions	Belin et al., 1998; Shears et al., 1998; Robertson et al., 2000; Benito-Sanz et al., 2017	MLPA or targeted deletion/duplication analysis; array-CGH/SNP array for larger PAR1 CNVs; karyotype/FISH if sex-chromosome rearrangement or mosaicism is suspected
Coding-region sequence variants (homeodomain)	4.2	Missense substitutions affecting DNA binding or nuclear localization signal within the homeodomain	R153C; V163F; R168W; R173C; A170P	Zinn et al., 2002; Shears et al., 2002; Sabherwal et al., 2004a; Ogata et al., 2002; Marchini et al., 2007b; Barca-Tierno et al., 2011	Sanger sequencing or validated NGS of SHOX coding exons and exon-intron boundaries; confirm by Sanger when needed
Frameshift/ truncating variants	4.2	Premature termination leading to nonsense-mediated mRNA decay or truncated/non-functional SHOX protein, depending on variant context	p.Ser16AlafsTer60; R118fsX130; P243fsX321	Zinn et al., 2002; Gürsoy et al., 2020	Sanger sequencing or validated NGS; deletion/duplication testing only when the truncating mechanism is a CNV rather than a sequence variant
Regulatory/enhancer deletions	4.3	Loss/disruption of conserved regulatory elements upstream or downstream of SHOX, presumed to reduce effective SHOX expression	240–350 kb 3′ deletion; 1.1 Mb downstream enhancer deletion; 249 kb downstream deletion	Fukami et al., 2005; Bertorelli et al., 2007; Campos-Barros et al., 2007; Bunyan et al., 2013	MLPA with upstream/downstream enhancer probes; array-CGH/SNP array; targeted qPCR or breakpoint sequencing for confirmation. Standard NGS should not be relied on unless PAR1/enhancer CNV coverage is validated
Splice-site variants	4.4	Predicted disruption of canonical splicing, likely causing exon skipping, intron retention, or aberrant transcripts	c.486 + 2T>C	Kohkalani et al., 2025	Sanger sequencing or validated NGS including splice junctions; RNA or minigene analysis can be considered when the effect is uncertain
Compound heterozygous configurations	4.1–4.4	Two different SHOX-inactivating events on opposing alleles	Deletion + missense; deletion + frameshift; SHOX deletion + enhancer deletion; cytogenetic rearrangement + enhancer deletion	Zinn et al., 2002; Ogata et al., 2002; Fukami et al., 2005; Campos-Barros et al., 2007; Tsuchiya et al., 2014	Combined testing is usually required: MLPA/array-CGH for CNVs plus Sanger/NGS for sequence variants; parental segregation, karyotype/FISH if sex-chromosome rearrangement or mosaicism is suspected

A170D was not included among representative LMD-causing variants because it has been described in heterozygous LWD, families, not as a directly confirmed homozygous LMD-causing variant in the cases reviewed here.

On partial SHOX, rearrangements, Benito-Sanz et al. (2017) reported intron 3 breakpoints in 12/28 (42.8%) partial deletions/duplications in their cohort and in 13/21 (62%) partial deletions and 23/38 (60.5%) partial duplications in their broader literature review.

Abbreviations: LMD, langer mesomelic dysplasia; LWD, Léri-Weill dyschondrosteosis; SHOX, short stature homeobox gene; NLS, nuclear localization signal; mRNA, messenger RNA.

From a clinical-genetic standpoint, suspected *SHOX* deficiency should be approached with a phenotype-first workflow. Clinical suspicion should be raised by disproportionate short stature, increased sitting-height/height ratio, mesomelic shortening, Madelung deformity, prenatal mesomelia, or a family history compatible with pseudoautosomal inheritance. For most *SHOX*-suspected cases, first-line testing should include *SHOX*/PAR1 copy-number analysis (e.g., MLPA or array-CGH/SNP array) to detect whole-gene, partial-gene, or enhancer-region CNVs. If CNV testing is negative or a sequence-level defect remains plausible, *SHOX* sequencing by Sanger or validated NGS should be added for coding and splice-site variants. Karyotype and/or FISH should be performed in parallel when Turner syndrome, sex-chromosome rearrangement, or mosaicism is clinically suspected. Standard NGS should not be used as the sole test for *SHOX*/PAR1 enhancer CNVs unless the assay includes analytically validated coverage and CNV calling for these regions. If targeted testing remains negative despite a strong phenotype, parental segregation studies and broader skeletal-dysplasia panel or exome/genome testing should be considered (Capkova et al., 2020; Spurna et al., 2022).

**FIGURE 4:**
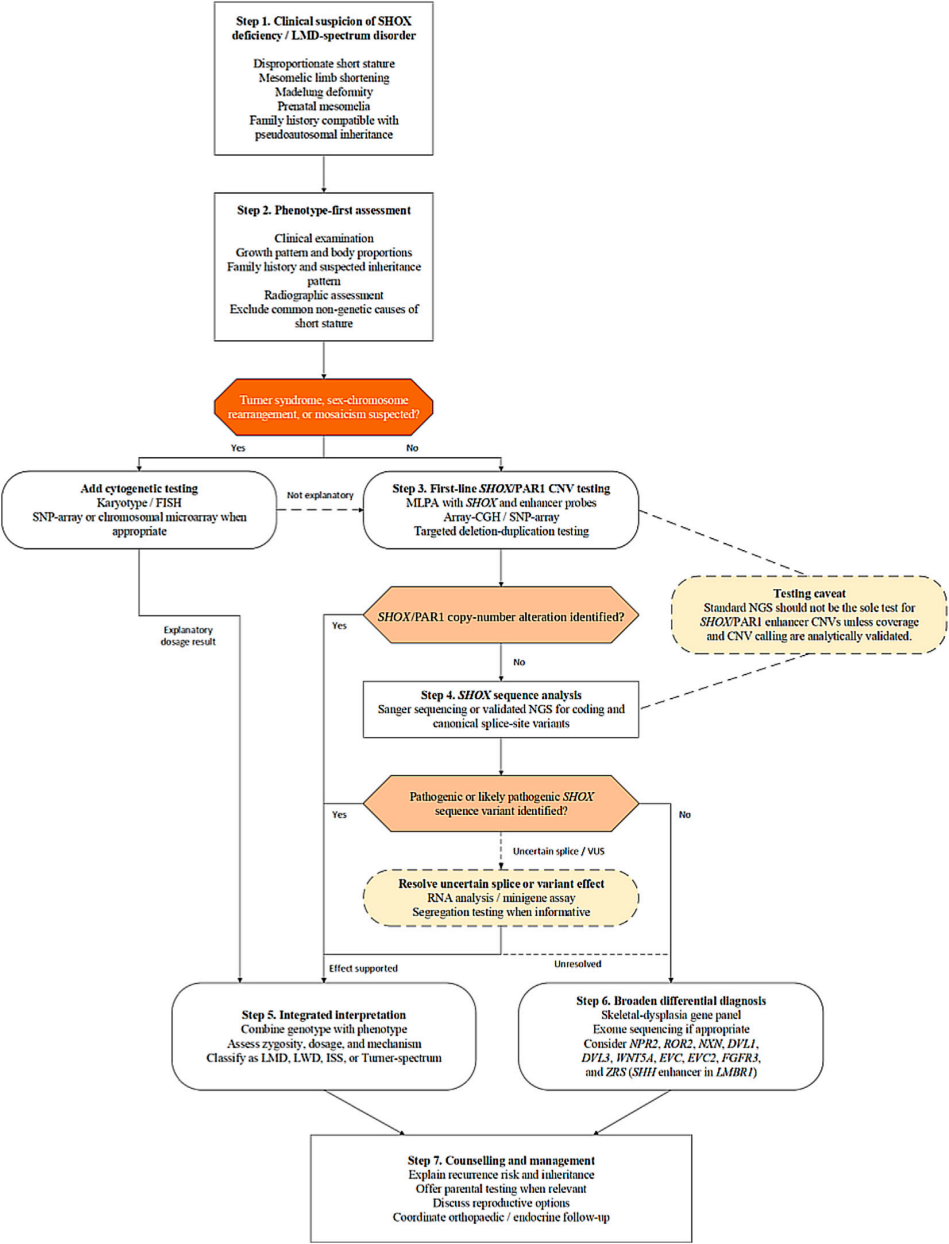
Decision-oriented clinical-genetic workflow for suspected *SHOX* deficiency and Langer mesomelic dysplasia-spectrum phenotypes. The workflow starts with phenotype-first assessment and then directs testing according to the most likely mechanism: *SHOX*/PAR1 copy-number analysis is proposed as first-line testing in most *SHOX*-suspected cases to detect whole-gene, partial-gene, or enhancer CNVs; *SHOX* sequence analysis is added when CNV testing is negative or a sequence-level defect is suspected; and cytogenetic evaluation is performed in parallel when Turner syndrome, sex-chromosome rearrangement, or mosaicism is clinically suspected. The dashed caveat highlights that standard NGS should not be used as the sole test for *SHOX*/PAR1 enhancer CNVs unless coverage and CNV calling for these regions are analytically validated. If no causative *SHOX*/PAR1 variant, CNV, or sex-chromosome dosage alteration is identified, broader skeletal-dysplasia panel testing or exome/genome sequencing may be considered. Confirmed or suspected molecular diagnoses should be followed by integrated genotype-phenotype interpretation, genetic counselling, recurrence-risk assessment, and discussion of reproductive options. Abbreviations: array-CGH, array comparative genomic hybridization; CNV, copy-number variant; FISH, fluorescence *in situ* hybridization; ISS, idiopathic short stature; LMD, Langer mesomelic dysplasia; LWD, Léri-Weill dyschondrosteosis; MLPA, multiplex ligation-dependent probe amplification; NGS, next-generation sequencing; NXN, nucleoredoxin; PAR1, pseudoautosomal region 1; RNA, ribonucleic acid; SHOX, short stature homeobox; SNP-array, single-nucleotide polymorphism array; VUS, variant of uncertain significance; ZRS, zone of polarizing activity regulatory sequence.

Figure 4 presents a decision-oriented clinical workflow for suspected *SHOX* deficiency and for the differential diagnosis of mesomelic dysplasias.

## 5 Differential diagnosis

The differential diagnosis of LMD encompasses a heterogeneous group of mesomelic and acromesomelic dysplasias that share the feature of predominant involvement of the middle segments of the limbs (Roth et al., 1996; Gahunia et al., 2009; Ambrosetti et al., 2014). Accurate distinction among these entities is essential for genetic counseling, reproductive planning, and prognostic assessment, as each condition carries a distinct mode of inheritance, recurrence risk, and pattern of extraskeletal involvement (Ambrosetti et al., 2014).

LWD, the heterozygous form of *SHOX* deficiency, represents the closest diagnostic consideration. LWD is characterized by milder disproportionate short stature, Madelung deformity, and mesomelic limb shortening without the severe ulnar and fibular aplasia/hypoplasia seen in LMD (Roth et al., 1996; Gahunia et al., 2009; Ambrosetti et al., 2014). Reinhardt-Pfeiffer mesomelic dysplasia, historically considered a separate entity, is now regarded as allelic to LWD (Ambrosetti et al., 2014).

Nievergelt mesomelic dysplasia is an autosomal dominant condition distinguished by a characteristic rhomboid or triangular shape of the tibia and fibula, as well as multiple radioulnar, tarsal, and carpal synostoses, features not observed in LMD (Roth et al., 1996; Gahunia et al., 2009; Ambrosetti et al., 2014).

Robinow syndrome (both autosomal dominant and recessive forms) presents with mesomelic shortening predominantly of the upper limbs, but is further characterized by distinctive craniofacial dysmorphism (“fetal face”), genital hypoplasia, and vertebral segmentation defects. Cognitive delay may occur in a minority of cases, whereas cognition is usually preserved in LMD (Ambrosetti et al., 2014; Roifman et al., 2015).

Ellis-van Creveld syndrome is characterized by shortening of the middle segments and extremities, post-axial polydactyly, dental abnormalities, a narrow thorax, and congenital heart defects, in contrast to LMD, in which no visceral abnormalities have been reported (Roth et al., 1996).

Werner-type mesomelic dysplasia (tibial hemimelia with polydactyly) is distinguished by tibial aplasia or hypoplasia with preserved or duplicated fibula, polydactyly, and absent thumbs. LMD primarily affects the ulna and fibula while sparing the digits (Roth et al., 1996; Gahunia et al., 2009; Ambrosetti et al., 2014).

The acromesomelic dysplasias, including the Maroteaux and Campailla-Martinelli types, should also be considered. The Maroteaux type presents with an enlarged head and disproportionately small hands and feet, while the Campailla-Martinelli type features marked forearm shortening with kyphoscoliosis. Both conditions differ from LMD in their pattern of skeletal segment involvement and the presence of acral abnormalities (Roth et al., 1996; Gahunia et al., 2009; Ambrosetti et al., 2014).

Hypochondroplasia, an autosomal dominant skeletal dysplasia usually caused by heterozygous pathogenic *FGFR3* variants, may also enter the differential diagnosis, particularly in milder or atypical presentations. Both hypochondroplasia and *SHOX*-related conditions feature disproportionate short stature, and mild cases of LWD lacking overt Madelung deformity can be difficult to distinguish clinically from hypochondroplasia (Thomas et al., 2004; Bober et al., 1999).

Finally, prenatal differential diagnosis may be particularly challenging, because fetal skeletal development in the first trimester has not yet progressed to the stage at which the classical radiographic and morphological features of LMD become apparent. Phenotypic heterogeneity within LMD and morphological overlap with other mesomelic dysplasias add further complexity. In such cases, a combined approach integrating family history, parental phenotyping, and molecular genetic testing is essential for definitive diagnosis (Thomas et al., 2004; Mazza et al., 2011; Ambrosetti et al., 2014).

Table 6 summarises the key distinguishing features of LMD versus the principal differential diagnoses discussed above.

**TABLE 6:** Differential diagnosis of Langer mesomelic dysplasia (LMD): key distinguishing features.

Condition	Inheritance	Pattern of limb shortening	Distinguishing features (vs. LMD)	References	Principal gene/molecular cause
LMD (reference)	Pseudoautosomal recessive (biallelic SHOX inactivation)	Severe mesomelic (forearms and lower legs)	Hypoplasia/aplasia of the ulna and fibula; visceral abnormalities generally absent; cognition usually preserved	Langer, 1967; Roth et al., 1996; Zinn et al., 2002	*SHOX*: biallelic coding, splice-site, whole-gene, PAR1, or enhancer-region loss-of-function
Léri-Weill dyschondrosteosis (LWD)	Pseudoautosomal dominant (heterozygous SHOX deficiency)	Mild to moderate mesomelic	Madelung deformity; milder phenotype without severe ulnar/fibular aplasia	Belin et al., 1998; Shears et al., 1998; Roth et al., 1996; Gahunia et al., 2009; Ambrosetti et al., 2014	*SHOX*: heterozygous coding, splice-site, CNV, or enhancer-region defect
Reinhardt-Pfeiffer mesomelic dysplasia^a^	Autosomal dominant	Mesomelic	Ulnar and fibular hypoplasia without other bone abnormalities; included within or overlapping the LWD spectrum in modern nosology, possibly allelic to LWD/LMD	Roth et al., 1996; Ambrosetti et al., 2014	Likely *SHOX*-related/allelic to the LWD spectrum; historical entity with limited molecular confirmation
Nievergelt syndrome	Autosomal dominant	Mesomelic	Rhomboid/triangular shape of tibia and fibula; multiple radioulnar, tarsal and carpal synostoses	Roth et al., 1996; Gahunia et al., 2009; Ambrosetti et al., 2014	Causative gene not consistently established in the classic Nievergelt phenotype
Robinow syndrome	Autosomal dominant or autosomal recessive	Mesomelic, predominantly upper limbs	Distinctive craniofacial dysmorphism (“fetal face”); genital hypoplasia; vertebral segmentation defects; cognitive delay may occur in a minority of cases	Roth et al., 1996; Gahunia et al., 2009; Ambrosetti et al., 2014; Roifman et al., 2015	AD: *DVL1*, *DVL3*, *WNT5A*; AR/Robinow-like: *ROR2*, *NXN*; WNT/PCP pathway genes
Ellis-van Creveld syndrome	Mostly autosomal recessive; rare autosomal dominant PRKACA/PRKACB-related forms	Mesomelic with acral involvement	Postaxial polydactyly; dental abnormalities; narrow thorax; congenital heart defects	Roth et al., 1996; Gahunia et al., 2009; Da Silva et al., 2023	*EVC*/*EVC2* and other *EVC*-spectrum ciliary or hedgehog-pathway genes, including DYNC2H1, DYNC2LI1, *GLI1*, *SMO*, *WDR35*, and rare *PRKACA*/*PRKACB*-related forms
Werner mesomelic dysplasia	Autosomal dominant	Mesomelic, predominantly tibia	Tibial hypoplasia/aplasia with preserved or duplicated fibula; polydactyly; absent thumbs; cleft lip and congenital heart defects may occur	Roth et al., 1996; Gahunia et al., 2009; Ambrosetti et al., 2014; Wieczorek et al., 2010	ZRS limb enhancer of *SHH* located in intron 5 of *LMBR1*
Acromesomelic dysplasia, Maroteaux type	Autosomal recessive	Acromesomelic	Postnatal growth failure; short/broad metacarpals and phalanges; primarily skeletal involvement	Roth et al., 1996; Bartels et al., 2004	*NPR2*/NPR-B: biallelic loss-of-function
Acromesomelic dysplasia, Campailla-Martinelli type	Autosomal recessive	Acromesomelic	Marked forearm shortening with hypoplastic fibula and kyphoscoliosis	Roth et al., 1996	Molecular basis not firmly established for the classic Campailla-Martinelli type
Hypochondroplasia^b^	Autosomal dominant (heterozygous FGFR3 pathogenic variants)	Mild short-limb disproportion; may mimic mild SHOX-related disproportion	Disproportionate short stature without severe Madelung deformity; can be clinically difficult to distinguish from mild LWD	Thomas et al., 2004; Bober et al., 1999	*FGFR3* heterozygous pathogenic variants

Differentiating features and inheritance patterns are primarily compiled from Roth et al. (1996) Table 1, Gahunia et al. (2009), and Ambrosetti et al. (2014). The distinguishing features of LMD are taken from Langer (1967) and Zinn et al. (2002).

Abbreviations: LMD, langer mesomelic dysplasia; LWD, Léri-Weill dyschondrosteosis; SHOX, short stature homeobox gene; FGFR3, fibroblast growth factor receptor 3; WNT/PCP, Wnt/planar cell polarity. The gene column lists the principal currently recognized molecular causes and is intended as a practical guide rather than an exhaustive gene panel.

^a^ Also referred to as Pfeiffer-Reinhardt mesomelic dysplasia in some sources.

^b^ Hypochondroplasia is included as a diagnostic pitfall for mild heterozygous SHOX-related phenotypes rather than as a classic differential diagnosis of severe LMD.

## 6 Summary

LMD is caused by biallelic or functionally complete SHOX deficiency resulting from pathogenic alterations in PAR1, including whole-gene or partial SHOX deletions, upstream or downstream enhancer-region CNVs, and intragenic missense, truncating, or splice-site variants. These findings highlight the need for comprehensive genetic testing using complementary molecular and cytogenetic techniques, as well as careful genetic counselling for affected families. In practice, severe mesomelic dysplasia should prompt coordinated SHOX/PAR1 CNV analysis, SHOX sequencing, and cytogenetic testing when sex-chromosome rearrangement or mosaicism is plausible, followed by broader skeletal-dysplasia testing if SHOX studies do not explain the phenotype. The marked phenotypic variability observed even among individuals carrying identical SHOX alterations suggests the contribution of additional genetic, epigenetic, or regulatory modifiers influencing disease severity. Current evidence also indicates that the overall functional dosage of SHOX, rather than variant class alone, plays a central role in determining the clinical phenotype across the SHOX-deficiency spectrum. Advances in molecular diagnostic methods, particularly MLPA and array-CGH, have substantially improved the detection of cryptic enhancer-region abnormalities and complex rearrangements within PAR1. Improved understanding of these molecular mechanisms may contribute to earlier diagnosis, more accurate prenatal assessment, and refined genotype-phenotype correlations in affected families.

## Glossary

array-CGH: array Comparative Genomic Hybridization
BNP: B-type natriuretic peptide
CNV: copy-number variant
DVL1/DVL3: Dishevelled Segment Polarity Protein 1/3
EVC/EVC2: Ellis-van Creveld syndrome genes 1/2
FGFR3: Fibroblast Growth Factor Receptor 3
FISH: fluorescence *in situ* hybridization
GH: growth hormone
IGF-1: insulin-like growth factor 1
ISS: idiopathic short stature
LMBR1: limb development membrane protein 1
LMD: Langer mesomelic dysplasia
LWD: Léri-Weill dyschondrosteosis
MLPA: Multiplex Ligation-dependent Probe Amplification
NGS: next-generation sequencing
NLS: nuclear localization signal
NPPB: natriuretic peptide B (gene)
NPR2: natriuretic peptide receptor 2
NXN: nucleoredoxin
OMIM: Online Mendelian Inheritance in Man
PAR1: pseudoautosomal region 1
qPCR: quantitative polymerase chain reaction
ROR2: receptor tyrosine kinase-like orphan receptor 2
SHH: Sonic Hedgehog
SHOX: Short Stature Homeobox-containing gene
SHOX2: Short Stature Homeobox 2
SNP: single-nucleotide polymorphism
SOX5: SRY-Box Transcription Factor 5
SOX6: SRY-Box Transcription Factor 6
SOX9: SRY-Box Transcription Factor 9
WNT5A: Wnt Family Member 5A
ZRS: zone of polarizing activity regulatory sequence
